# Fuzzy Logic Based Control for Autonomous Mobile Robot Navigation

**DOI:** 10.1155/2016/9548482

**Published:** 2016-09-05

**Authors:** Hajer Omrane, Mohamed Slim Masmoudi, Mohamed Masmoudi

**Affiliations:** Department of Electrical Engineering, METS Laboratory, National School of Engineers of Sfax, University of Sfax, Street of Soukra Km. 4, BP 1173, 3038 Sfax, Tunisia

## Abstract

This paper describes the design and the implementation of a trajectory tracking controller using fuzzy logic for mobile robot to navigate in indoor environments. Most of the previous works used two independent controllers for navigation and avoiding obstacles. The main contribution of the paper can be summarized in the fact that we use only one fuzzy controller for navigation and obstacle avoidance. The used mobile robot is equipped with DC motor, nine infrared range (IR) sensors to measure the distance to obstacles, and two optical encoders to provide the actual position and speeds. To evaluate the performances of the intelligent navigation algorithms, different trajectories are used and simulated using MATLAB software and SIMIAM navigation platform. Simulation results show the performances of the intelligent navigation algorithms in terms of simulation times and travelled path.

## 1. Introduction

Robotics is an important area of research that uses knowledge across several disciplines such as mechanics, electronics, and computer engineering, to move a mobile robot in defined environment with some degree of autonomy.

One of the major challenges of the autonomous navigation for mobile robots is the detection and obstacles avoidance during the robot navigation task. This problem can be solved by relating different methods or algorithms in order to attain best results.

Fuzzy logic is used in the design of possible solutions to perform local navigation, global navigation, path planning, steering control, and rate control of a mobile robot [1].

Many research literatures used soft computer algorithms to control mobile robots in academic field as well as in the engineering field.

Yu et al. used Taguchi method to design an optimized fuzzy logic controller for trajectory tracking of a wheeled mobile robot [2].

Xiong and Qu elaborated a method for intelligent vehicles path tracking with two fuzzy controllers combining with vehicle control direction [3].

Selekwa et al. detailed the design of a preference-based fuzzy behaviour system for navigating robotic vehicles employing the multivalued logic framework. The proposed technique permits the robot to effectively navigate through cluttered environments [4].

Jia et al. developed a method of fuzzy control of the smart tennis chair using pressure sensors and omnidirectional wheels. The wheelchair can be master of a manual way with joystick device [5].

For this paper, we developed a fuzzy controller for the mobile robot navigation with different desired positions. Then, a second intelligent controller based on fuzzy logic has been developed to achieve the navigation and obstacle avoidance tasks. The latter is provided by infrared range (IR) sensors and the discrete kinematic model.

Zero-order Sugeno model is used to develop different fuzzy logic controllers. The implication method is simply multiplication, and the aggregation operator just includes all of the singletons.

This paper is organized into seven sections. In Section 2 the mathematical model of the robot is presented.

In Section 3, the fuzzy logic controller is developed and discussed in detail. In Section 4 simulation results show the efficiency of the proposed method. In Section 5, we describe the fuzzy logic controller to avoid obstacles.  Section 6 presents the results of the simulation and experimental tests.

Finally, the conclusions based on the obtained results are given in Section 7.

## 2. The Kinematic Model of the Mobile Robot

**Figure 1:**
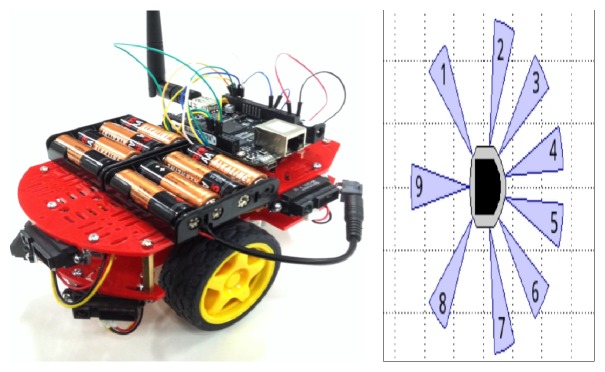
QuickBot robot and SIMIAM simulator model.

Our mobile robot model is a unicycle robot type. It has two degrees of freedom and moved with two DC motors. The QuickBot is equipped with nine infrared range (IR) sensors, six are located in the front, and three are placed on its back. The robot has a two-wheel differential drive system moved by two DC motors equipped with optical encoder for each (Figure 1) [6].

The configuration of the mobile robot is characterized by the position (*X*, *Y*) and the orientation in a Cartesian coordinate.

**Figure 2:**
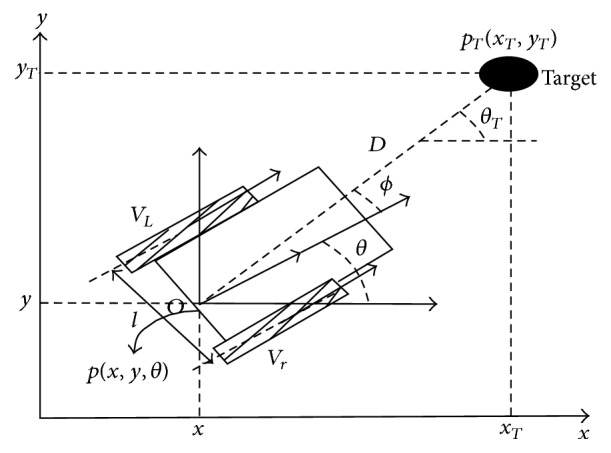
Kinematic model of the mobile robot.

Figure 2 shows the variables used in the kinematic model; it has several parameters that are as follows:

 
*V*_*R*_: linear velocity of the right wheel.
 
*V*_*L*_: linear velocity of the left wheel.
 
*W*: angular velocity of the mobile robot.
 
*X*: abscissa of the robot.
 
*Y*: intercept of the robot.
 
*X*, *Y*: the actual position coordinates.
 
*θ*: orientation of the robot.
 
*L*: the distance between the driving wheels.

The kinematic model [7] is given by these equations:

(1) dxdt=VL+VR2cos⁡θ,dydt=VL+VR2sin⁡θ,dθdt=VL−VR2,

where (*X*, *Y* and *θ*) are the robot actual position and orientation angle in world reference frame. In simulation, we use the discrete form to build a model of the robot. These equations are used to simulate the robot in MATLAB software. Then, the discrete form of the kinematic model is given by the following equations:

(2) Xk+1=Xk+TVrk+Vlk2cos⁡θk,Yk+1=Yk+TVrk+Vlk2sin⁡θk,θk+1=θk+TVrk+VlkL,

where *X*_*k*+1_ and *Y*_*k*+1_ represent the position of the center axis of the mobile robot and* T* is the sampling time.

## 3. Design of the Fuzzy Logic Controller (FLC)

The developed fuzzy controller manages at the same time navigation and obstacle avoidance tasks. Many academic studies propose the fuzzy logic theory as a solution to control mobile robots [8–11]. The basic structure of the fuzzy controller is composed of three blocks: the fuzzification, inference, and defuzzification.

The first step to realize a fuzzy controller is fuzzification which transforms each real value's inputs and outputs into grades of membership for fuzzy control terms. The second part is fuzzy inference which combines the facts acquired from the fuzzification of the rule base and conducts the fuzzy reasoning process. There are some methods of fuzzy inference depending on the uses and the form of the membership function. When the input and the output variables and membership function are defined, the fuzzy rule is presented as the following form:

 IF 〈antecedents〉 THEN 〈conclusions〉 rules.

The third part of fuzzy logic is defuzzification block. The objective of this part is to transform the subsets of the outputs which are calculated by the inference engine.

### 3.1. Fuzzy Logic Controller (FLC)

The developed fuzzy logic controller (FLC) for navigation task used two inputs: the distance *d* and the angle orientation *φ*. The outputs of the controller are the speed of the right *V*_*R*_ and the left wheels *V*_*L*_. The values of the two inputs are given by the following equations:

(3) d=XT−X2+YT−Y2,φ=θT−θ,

with

(4) $$\begin{array}{c} \theta_{T}=\tan^{-1}\frac{\left(Y_{T}-Y\right)}{\left(X_{T}-X\right)}. \end{array}$$

**Figure 3:**
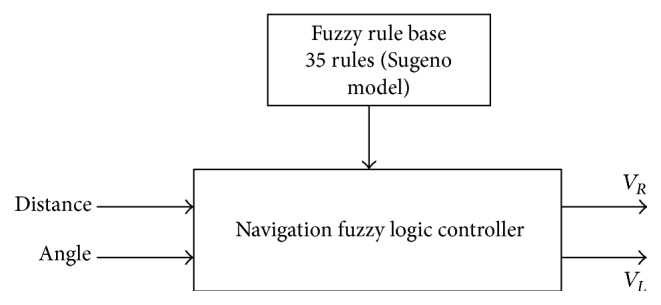
Methods of the conventional tracking fuzzy control system.

**Figure 4:**
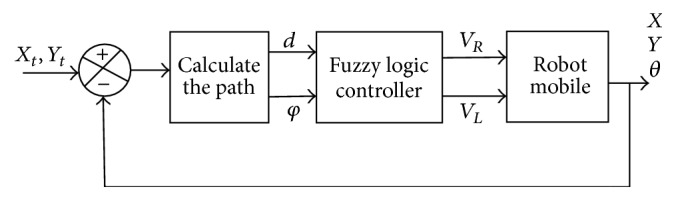
The block diagram of the robotic system.

Figure 3 illustrates the block diagram of the robotic system using a fuzzy controller to reach the desired position. The final intelligent control loop is described in Figure 4.

### 3.2. Structure of the Membership Function

Triangular and trapezoidal membership functions are used in the fuzzification block.

**Figure 5:**
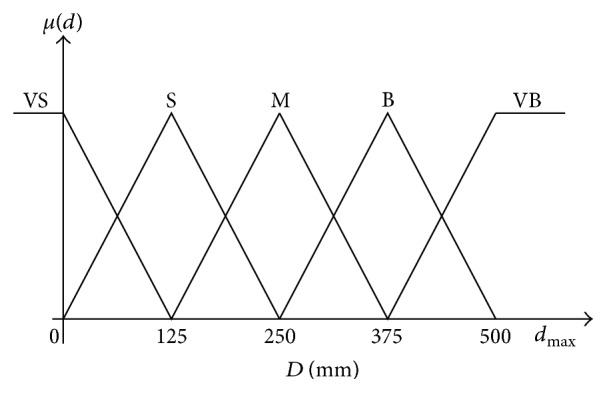
Input membership function for distance.

The variable *d* is partitioned in the universe of discourse [0 500 mm] which is defined by five triangular membership functions: very small (VS), small (S), medium (M), big (B), and very big (VB) as shown in Figure 5.

**Figure 6:**
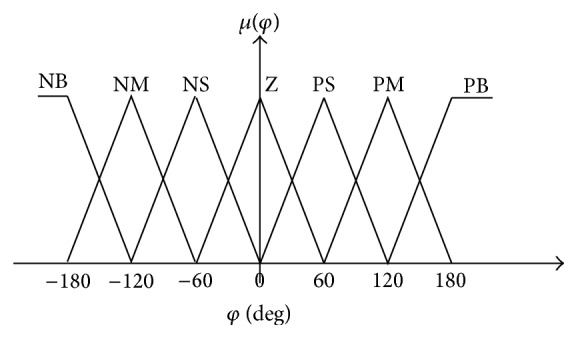
Input membership function for angle.

The angle should be partitioned in the universe of discourse [−180° 180°], and the setting is defined by seven membership functions: negative big (NB), negative medium (NM), negative small (NS), zero (Z), positive small (PS), positive medium (PM), and positive big (PB) as shown in Figure 6.

**Figure 7:**
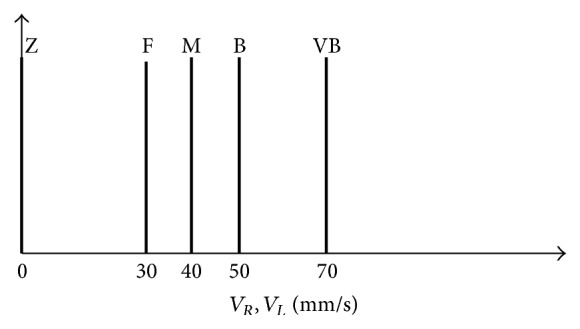
Output membership function for velocity.

For speed five singleton memberships functions are determined: Z (zero), F (far), M (medium), B (big), and VB (very big) as shown in Figure 7.

### 3.3. The Structure of the Fuzzy Rule Bases

To fulfill the control objective, it is crucial to design a fuzzy logic control for the real velocities of the mobile robot which use fuzzy control in the inputs and outputs. After detailing membership functions, we define the fuzzy rule bases. The expert system is established based on 35 IF-THEN rules. The fuzzy rule bases are presented in Table 1.

## 4. Simulation Results

In this part, we will present the results of our simulation system using MATLAB and SIMIAM simulator. The robot starts at the beginning position (*X*_0_, *Y*_0_), moving based on its wheels velocity, to reach its target position (*X*_*T*_, *Y*_*T*_).

Several tests, for different configurations of the desired positions, have been carried out.

In these simulations, we have considered that the mobile robot always starts from the same point (0, 0) and moved reaching different target positions (Figure 6).

**Figure 8:**
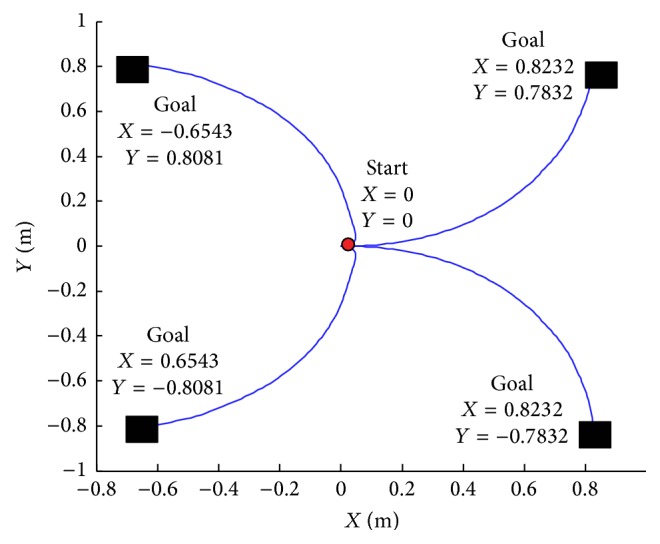
Navigation trajectories for different targets.

Figure 8 illustrates four trajectory positions.

The fuzzy controllers, previously described, have been tested with different situation. In Figure 8 we assume that the starting point is *X* = 0 and *Y* = 0 and the final point is *X*_*k*_ = 0.8232 and *Y*_*k*_ = −0.7832. It permits modifying the speed of the left and right wheel. This controller steers the robot to reach the target.

In Figure 10 we suppose that the starting point is *X* = 0 and *Y* = 0 and the final point is *X*_*k*_ = 0.8232 and *Y*_*k*_ = 0.7832.  Figure 11 shows the initial position from *X* = 0 and *Y* = 0 to *X*_*k*_ = 0.8232 and *Y*_*k*_ = 0.7832.

**Figure 9:**
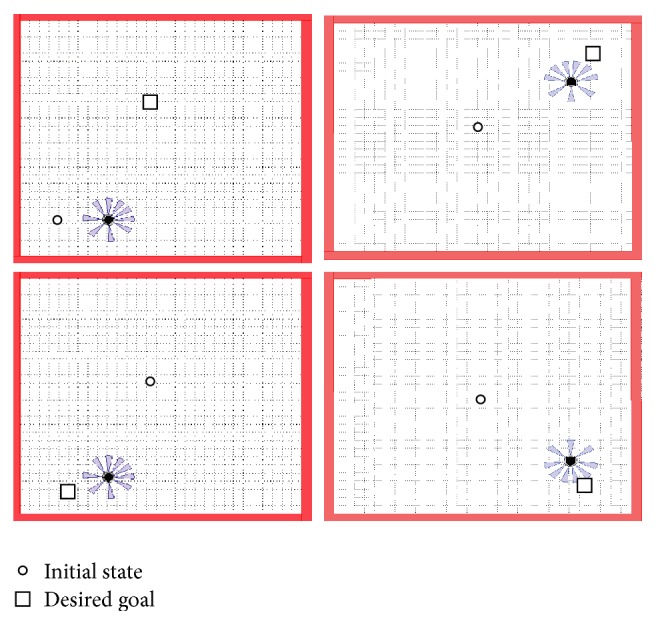
Simulation results for different target using SIMIAM platform.

**Figure 10:**
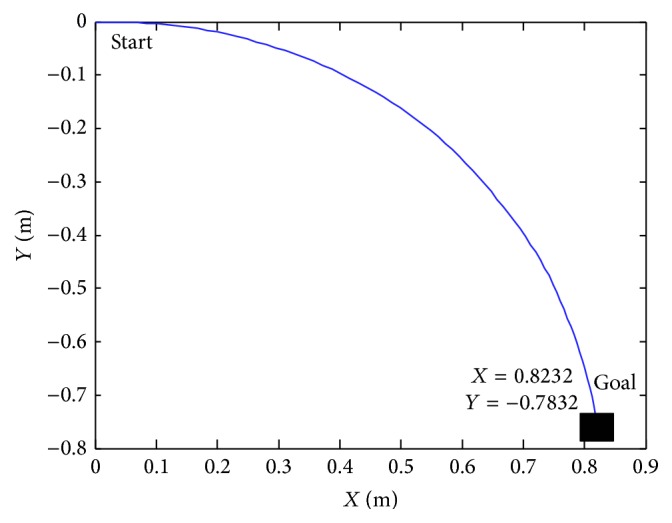
Navigation position with *X*_*k*_ = 0.8232 and *Y*_*k*_ = −0.7832.

**Figure 11:**
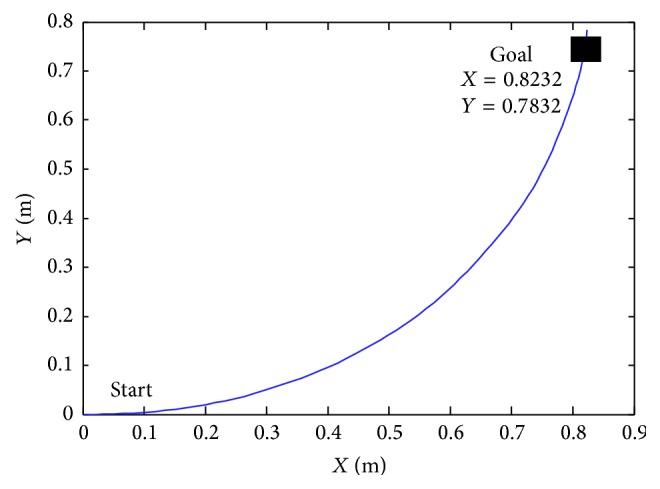
Navigation positions with *X*_*k*_ = 0.8232 and *Y*_*k*_ = 0.7832.

Figure 9 explains the navigation of the mobile robot in various positions using the MATLAB software.

**Figure 12:**
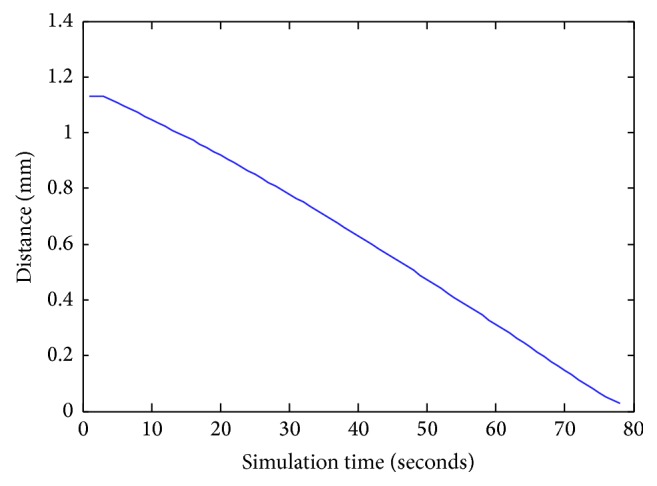
The distance *D* values during navigation.

**Figure 13:**
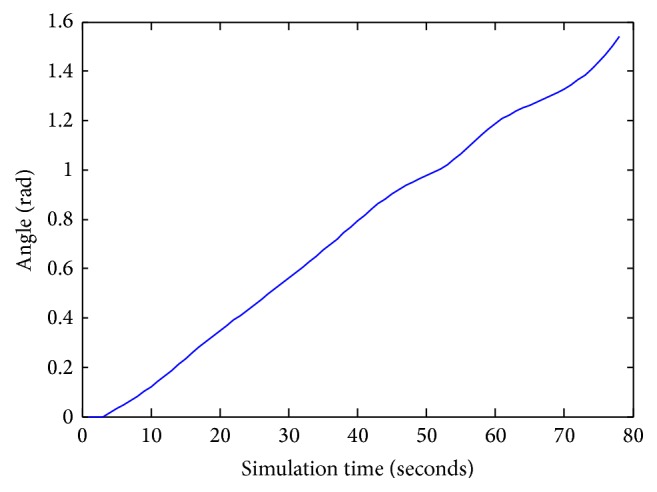
The angle *φ* values during navigation.

Figures 12 and 13 indicate the variation of angle orientation and distance at various simulation times without obstacles. At the starting simulation, the robot starts navigation by the distance of 1.22 mm. At 80 seconds, of simulation time, the robot attains the goal in the distance of 0.1 mm and the value of angle is 1.5 rad.

**Figure 14:**
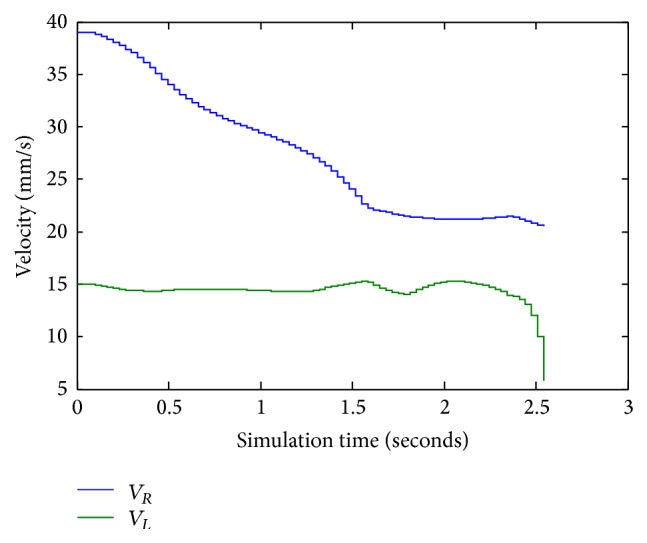
Left and right velocities values for *X*_*k*_ = 0.8232 and *Y*_*k*_ = −0.7832.

Figure 14 represents the right and the left velocity of the robot in simulation time. When the mobile robot has been oriented towards its target point, the FLC compute left and right robot wheel speeds to reach the goal.

## 5. Obstacle Avoidance Controller (OAC)

**Figure 15:**
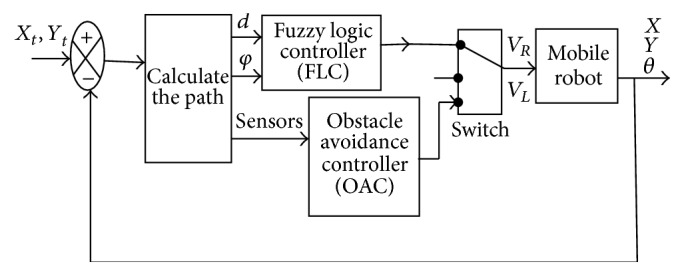
Block diagram with obstacle avoidance.

In this part, we used a control system based on two fuzzy behaviours with a single coordination unit for the choice between the two controllers: the first controller for convergence towards the goal and the second controller for obstacle avoidance using the infrared sensors.  Figure 15 illustrates the block diagram with obstacle avoidance.

The use of two fuzzy controllers consumes more resources and takes longer during navigating process of the mobile robot, so we optimize the configuration to use only one fuzzy controller for navigation and obstacle avoidance (FLCAO) (see Figure 22). Sugeno model is also used for its implementation performances.

**Figure 16:**
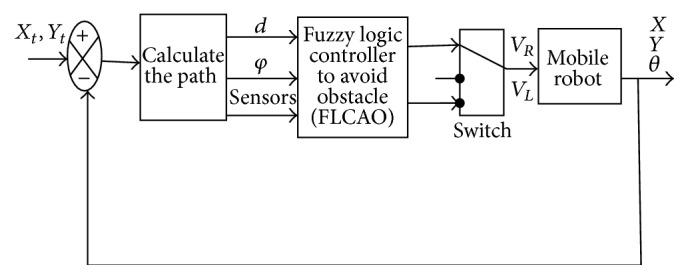
Block diagram with FLCAO.

**Figure 17:**
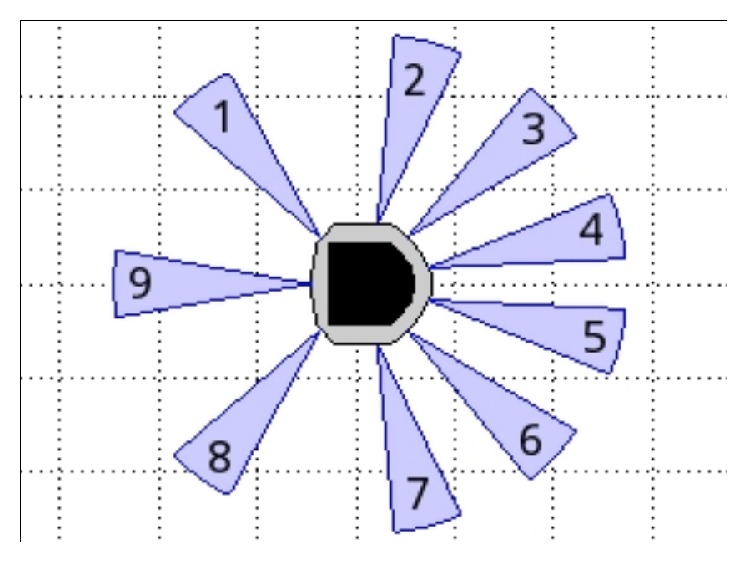
Infrared range (IR) sensor configuration.

Figure 16 shows the optimization of the configuration by using only one fuzzy controller for navigation and obstacles avoidance (FLCAO). SIMIAM robot is equipped with nine infrared sensors (Figure 17).

The range sensors are effective in the range 0.02 m to 0.2 m. However, the sensors return raw values in the range of [18; 3960] instead of the measured distances [12] (Figure 17).

### 5.1. Fuzzy Sets of the Input and Output

**Figure 18:**
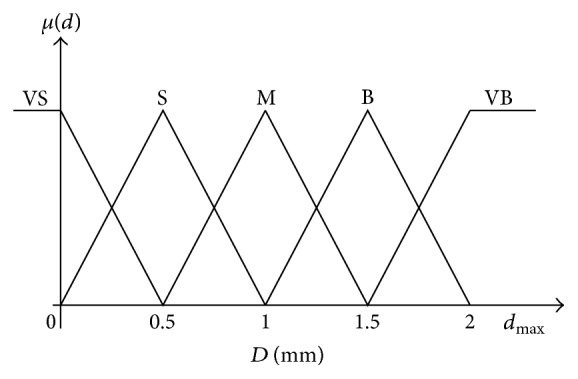
Input membership function for distance.

**Figure 19:**
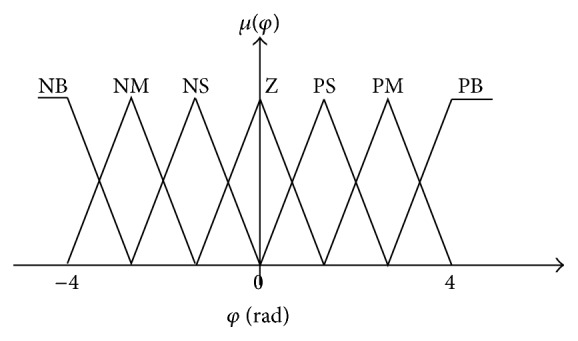
Input membership function for orientation angle.

**Figure 20:**
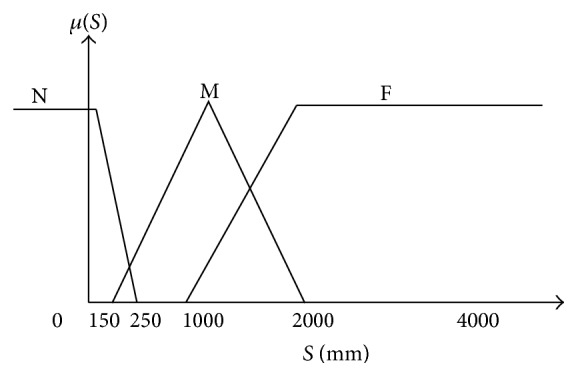
Input membership function for distance sensors.

There are three inputs to the fuzzy logic system and two outputs. The inputs are basically the distance (Figure 18), the angle orientation (Figure 19), and values of eight distance sensors given by S1, S2, S3, S4, S5, S6, S7, and S8 (Figure 20). Two outputs are generated: left velocity (*V*_*L*_) and right velocity (*V*_*R*_).

The variable *d* is partitioned in the universe of discourse [0 2 mm] which is defined by five triangular membership functions: very small (VS), small (S), medium (M), big (B), and very big (VB) as shown in Figure 18.

The angle should be partitioned in the universe of discourse [−4 4 rad], and the setting is defined by seven membership functions: negative big (NB), negative medium (NM), negative small (NS), zero (Z), positive small (PS), positive medium (PM), and positive big (PB) as shown in Figure 19.

The sensor is divided into three membership functions: N (Near), M (Medium) and F (Far) (Figure 20).

**Figure 21:**
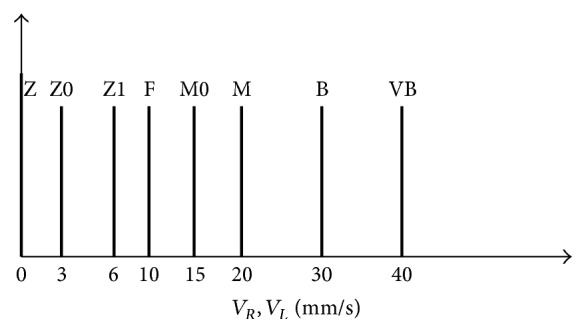
Input membership function for velocity.

**Figure 22:**
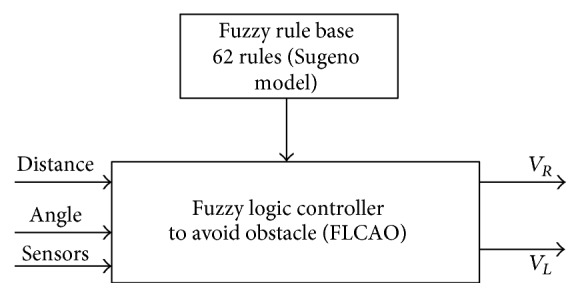
The FLCAO input/output configuration.

Figure 21 shows the eight membership functions: Z (zero), F (far), M (medium), B (big) and VB (very big) for the left and the right velocity (*V*_*R*_, *V*_*L*_).

The controller was a Sugeno-type fuzzy model defined with set of 62 fuzzy control rules. Some fuzzy rule bases are shown in the following:

 If (Distance is VS) and (ANgle is NB) and (S1 is F) and (S2 is F) and (S3 is F) and (S4 is F) then (*V*_*R*_ is B) ( *V*_*L*_ is 0)
 If (Distance is VS) and (ANgle is NM) and (S1 is F) and (S2 is F) and (S3 is F) and (S4 is F) then (*V*_*R*_ is M) ( *V*_*L*_ is 0)
 If (Distance is VS) and (ANgle is NB) and (S1 is F) and (S2 is F) and (S3 is F) and (S4 is F) then (*V*_*R*_ is F) (*V*_*L*_ is 0)
 ⋮
 If (S3 is M) and (S4 is M) then (*V*_*R*_ is M) (*V*_*L*_ is Z0)

## 6. Simulation Results with Obstacles Avoidance

In this section, the mobile robots have to navigate in environment with obstacles.

**Figure 23:**
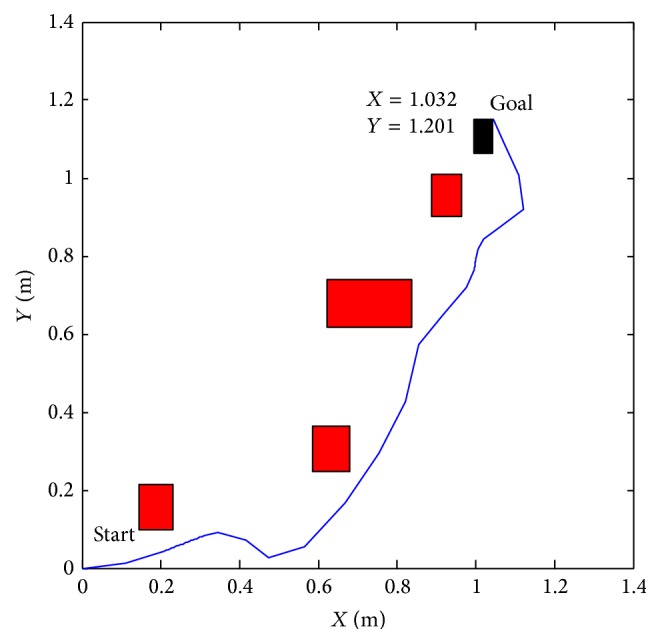
Reaching target with obstacles *X* = 1.032 and *y* = 1.201.

**Figure 24:**
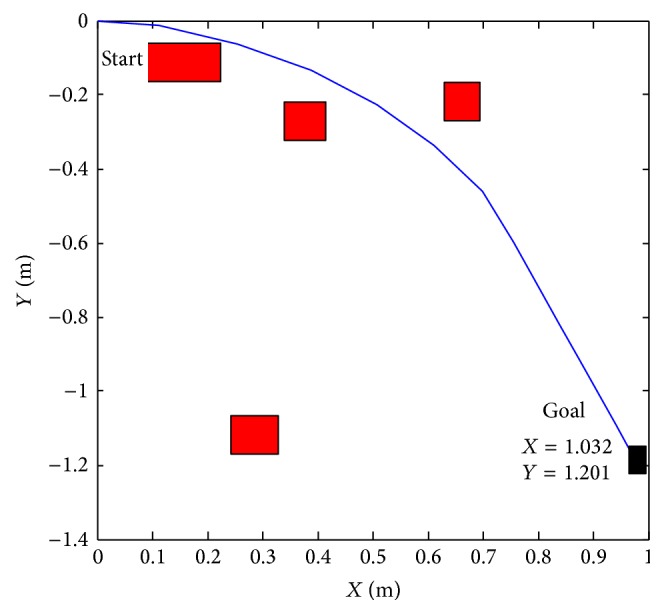
Reaching target with obstacles *X* = 1.032 and *y* = −1.201.

Figures 23 and 24 show that the proposed algorithm avoids the obstacle. We notice that if the infrared range sensors detect an obstacle, the robot is forced to make an adequate turn to avoid collision with the object.

Figures 25, 26, 27, 28, 29, 30, 31, and 32 show the distance sensor values. At the beginning of the simulation, the robot starts sensing the environment for possible obstacle detection.

**Figure 33:**
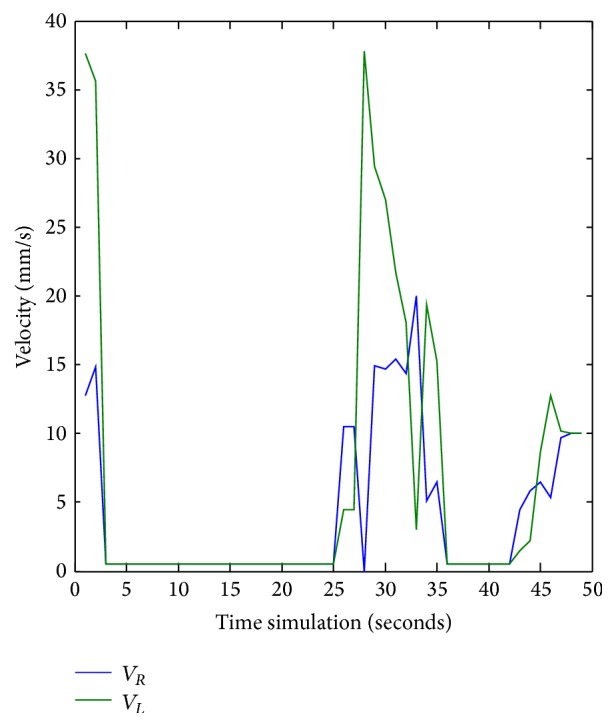
Left and right velocities for the robot during obstacle avoidance task. *X*_*k*_ = 0.8232 and *Y*_*k*_ = −0.7832.

**Figure 34:**
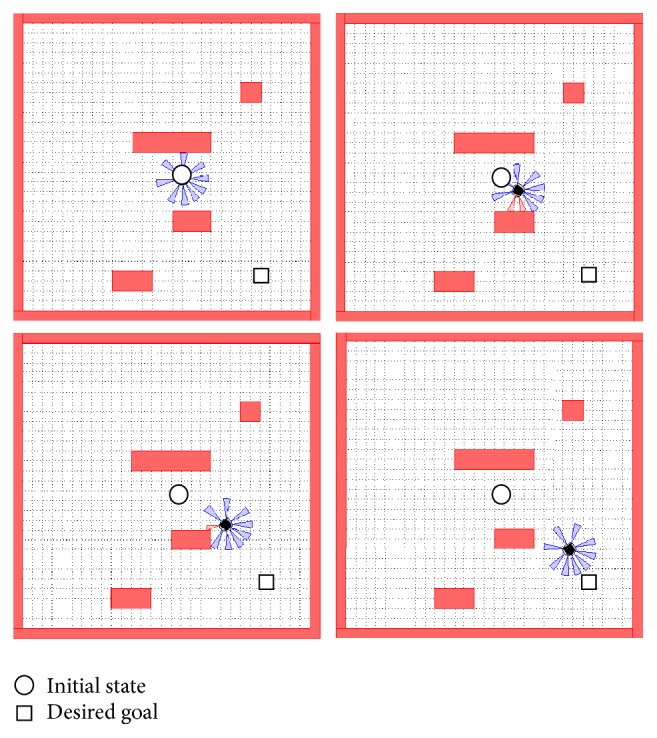
Sequence showing the MATLAB robot simulator SIMIAM implementing the navigation system with *X*_*k*_ = 0.8232 and *Y*_*k*_ = −0.7832.

Figure 33 shows the left and right wheels' velocities of the robot.  Figure 34 describes the navigation and obstacle avoidance of mobile robot in various positions using MATLAB.

At 25 seconds of the simulation time, sensors S3 reached a value of 1000.

At 32 seconds, the robot detects another obstacle on its right side and moves forward. Then, the robot gets stuck in between two obstacles and another obstacle in front of it.

Sensors S4 attain a value of 500.

Besides, the robot detects another obstacle on its path and turns left at 35 s; sensors S5 reach a value of 3750. Finally, at 42 s, the robot picks up another obstacle on its right side when sensors S1 attain 2750.

## 7. Conclusion

This paper presents the design and simulation of an intelligent mobile robot with encoders and infrared sensors detection computed with a navigation fuzzy control algorithm and obstacle avoidance system. First, we started with the development of the kinematic model of the robot system and then we designed the navigation fuzzy controller. The simulation results, using MATLAB and SIMIAM simulation platform, have shown the effectiveness of the designed FLC giving good navigation performances.

To add the obstacle avoidance task we opted for a second fuzzy controller. This solution seems to be effective; but since the final objective is the implementation on a dedicated embedded platform we chose using a single fuzzy controller which performed both tasks.

Simulation results using SIMIAM platform have shown a clear performance improvement of the navigation system. Thus, the fuzzy implemented controllers have shown its adaptation to the dynamics of unicycle robots. In fact, QuickBot is capable of reaching the target position and avoiding obstacles based on an optimized intelligent controller.

The results of the simulation and experimental tests in a real environment showed the success of the FLC algorithm in controlling QuickBot.

The final fuzzy logic controller can be implemented on an intelligent wheelchair to help the elderly or disabled people to perform the navigation task.

## Figures and Tables

**Figure 25:**
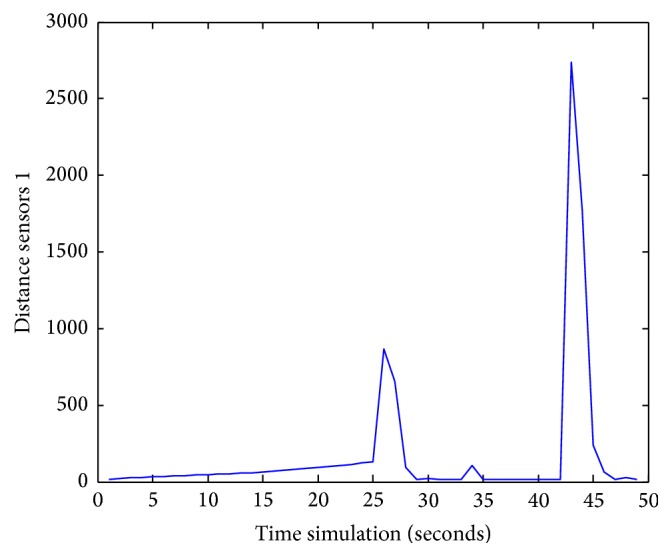
S1 values during obstacles avoidance with *X*_*k*_ = 0.8232 and *Y*_*k*_ = −0.7832.

**Figure 26:**
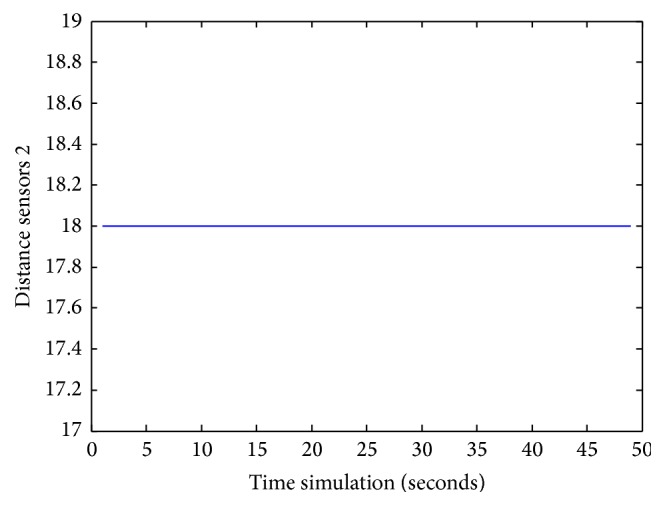
S2 values during obstacles avoidance.

**Figure 27:**
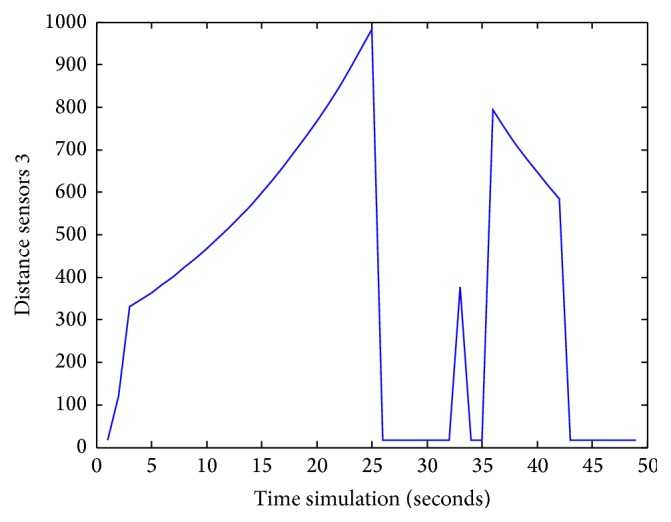
S3 values during obstacles avoidance.

**Figure 28:**
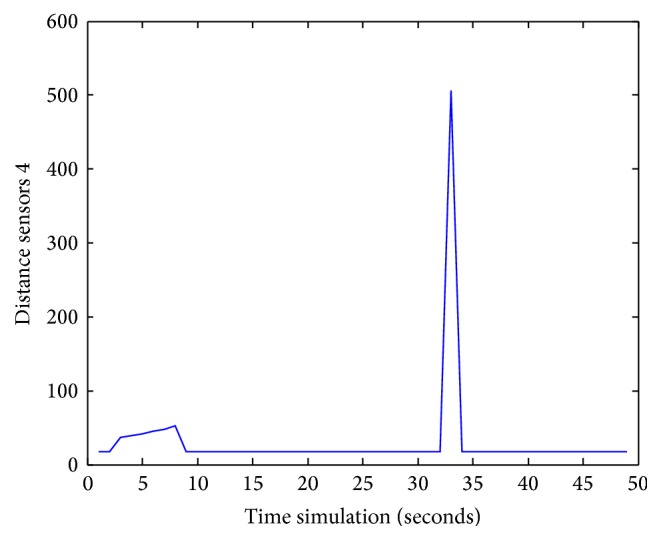
S4 values during obstacles avoidance.

**Figure 29:**
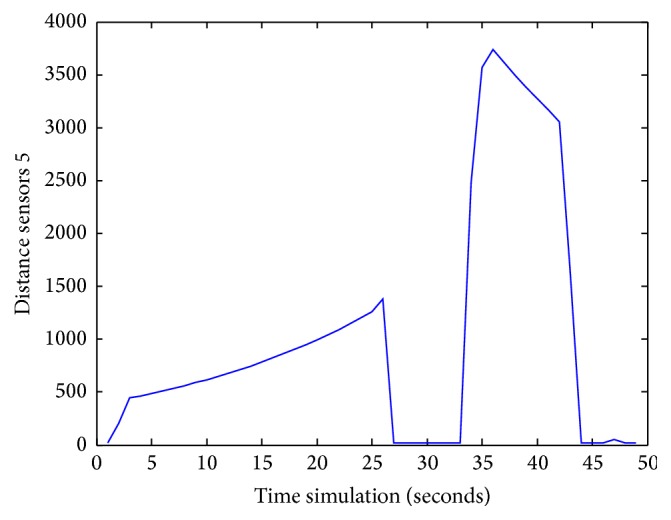
S5 values during obstacles avoidance.

**Figure 30:**
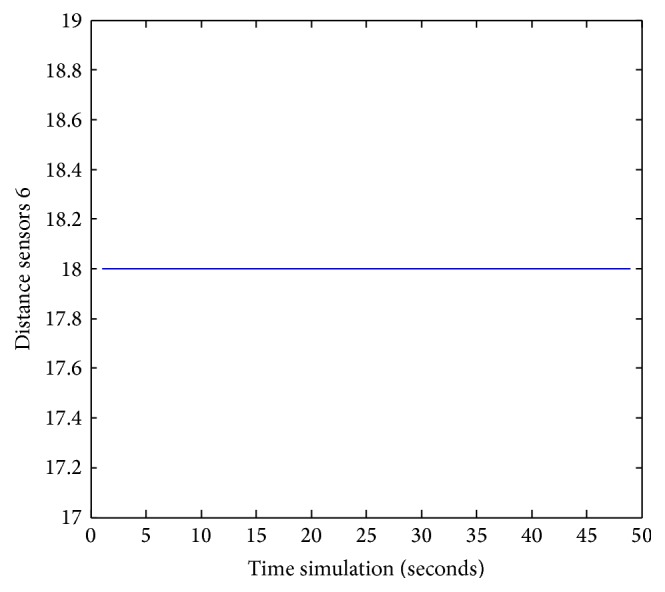
S6 values during obstacles avoidance.

**Figure 31:**
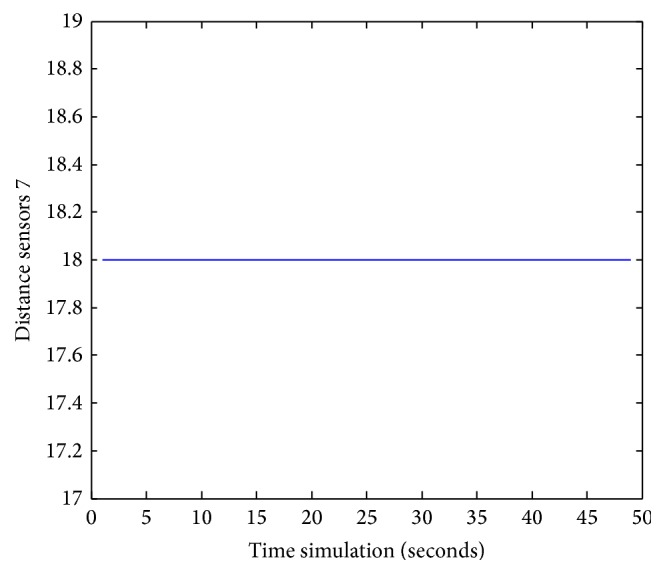
S7 values during obstacles avoidance.

**Figure 32:**
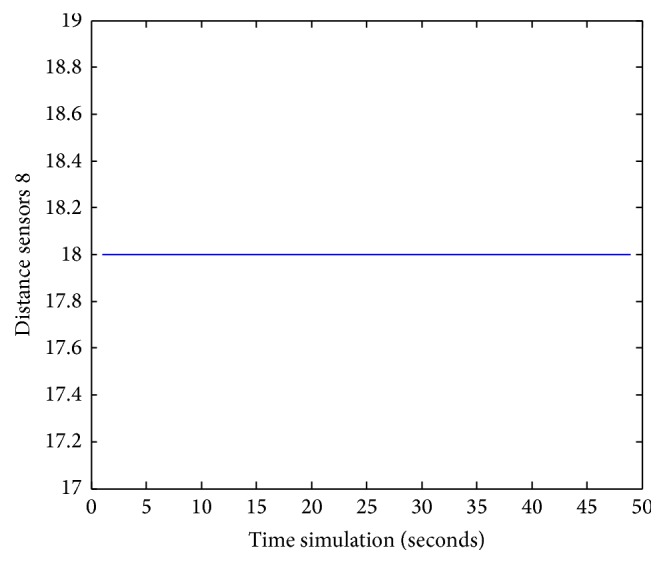
S8 values during obstacles avoidance.

**Table 1:** The fuzzy rule bases for the controller.

		Angle (*φ*)													
		NB		NM		NS		Z		PS		PM		PB	
		*V*_*R*_	*V*_*L*_	*V*_*R*_	*V*_*L*_	*V*_*R*_	*V*_*L*_	*V*_*R*_	*V*_*L*_	*V*_*R*_	*V*_*L*_	*V*_*R*_	*V*_*L*_	*V*_*R*_	*V*_*L*_
Distance (*D*)	VS	B	Z	M	Z	F	Z	S	F	Z	F	Z	F	Z	M
	S	VB	Z	B	Z	M	Z	S	F	Z	M	Z	B	Z	VB
	M	VB	Z	VB	Z	B	Z	M	M	Z	B	Z	VB	Z	VB
	B	VB	Z	VB	Z	VB	Z	B	B	Z	VB	Z	VB	Z	VB
	VB	VB	Z	VB	Z	VB	Z	VB	VB	Z	VB	Z	VB	Z	VB
